# Genomic characterisation and ecological distribution of *Mantoniella tinhauana*: a novel Mamiellophycean green alga from the Western Pacific

**DOI:** 10.3389/fmicb.2024.1358574

**Published:** 2024-05-07

**Authors:** Elvira Rey Redondo, Yangbing Xu, Charmaine Cheuk Man Yung

**Affiliations:** Department of Ocean Science, The Hong Kong University of Science and Technology, Kowloon, Hong Kong SAR, China

**Keywords:** biogeography, genomics, Mamiellophyceae, *Mantoniella tinhauana* sp. nov., marine algae, metagenomics

## Abstract

Mamiellophyceae are dominant marine algae in much of the ocean, the most prevalent genera belonging to the order Mamiellales: *Micromonas*, *Ostreococcus* and *Bathycoccus*, whose genetics and global distributions have been extensively studied. Conversely, the genus *Mantoniella*, despite its potential ecological importance, remains relatively under-characterised. In this study, we isolated and characterised a novel species of Mamiellophyceae, *Mantoniella tinhauana*, from subtropical coastal waters in the South China Sea. Morphologically, it resembles other *Mantoniella* species; however, a comparative analysis of the 18S and ITS2 marker genes revealed its genetic distinctiveness. Furthermore, we sequenced and assembled the first genome of *Mantoniella tinhauana*, uncovering significant differences from previously studied Mamiellophyceae species. Notably, the genome lacked any detectable outlier chromosomes and exhibited numerous unique orthogroups. We explored gene groups associated with meiosis, scale and flagella formation, shedding light on species divergence, yet further investigation is warranted. To elucidate the biogeography of *Mantoniella tinhauana*, we conducted a comprehensive analysis using global metagenomic read mapping to the newly sequenced genome. Our findings indicate this species exhibits a cosmopolitan distribution with a low-level prevalence worldwide. Understanding the intricate dynamics between Mamiellophyceae and the environment is crucial for comprehending their impact on the ocean ecosystem and accurately predicting their response to forthcoming environmental changes.

## Introduction

Mamiellophyceae, an early-branching class of photosynthetic picoeukaryotes within the Chlorophyta (Marin and Melkonian, 2010), dominate various ocean regions (Wang et al., 2019; Yung et al., 2022), particularly coastal waters (Lopes dos Santos et al., 2017; Tragin and Vaulot, 2018). Among the Mamiellophyceae, the order Mamiellales, which includes the families Mamiellaceae and Bathycoccaceae, encompasses the most extensively studied marine Mamiellophyceae genera: *Micromonas* (Mamiellaceae), *Ostreococcus* and *Bathycoccus* (Bathycoccaceae), and more recently, *Mantoniella* (Mamiellaceae).

Understanding the biogeography and diversity of Mamiellophyceae is crucial due to their important role in primary production, impact on ocean ecosystems and sensitivity to environmental disturbances (Monier et al., 2016). Distinguishing Mamiellophyceae species based on morphology alone is challenging, as evidenced by the similarities among previously identified *Mantoniella* species (Yau et al., 2020b). Therefore, molecular methods are essential for accurate species identification and unravelling the relationship between their distribution and environmental factors. Previous studies have revealed genetic adaptations and distinct ecological niches within different Mamiellophyceae clades and species (Lovejoy et al., 2007; Foulon et al., 2008; Demir-Hilton et al., 2011; Subirana et al., 2013; Hu et al., 2016; Simmons et al., 2016; Simon et al., 2017; Tragin and Vaulot, 2019). For example, *M. antarctica* (Marchant et al., 1989)*, M. baffinensis* and *M. beaufortii* (Balzano et al., 2012; Yau et al., 2020b) were exclusively found in cold, polar waters, while *M. squamata* is the only named *Mantoniella* species known to have a distribution beyond polar regions (Tragin and Vaulot, 2019; Belevich et al., 2021).

The predominant approach in Mamiellophyceae biogeography studies is metabarcoding, which involves sequencing marker genes such as the hypervariable regions of 18S ribosomal RNA (rRNA) (Monier et al., 2016; Tragin and Vaulot, 2019; Belevich et al., 2021). Metabarcoding has inherent limitations in true quantification and suffers from biases stemming from PCR recovery, strain polymorphisms, gene copy number variations and sequencing artifacts (Monier et al., 2016), and can underestimate the diversity of microbial eukaryotes (Piganeau et al., 2011a). For instance, *Bathycoccus* 18S rRNA sequences were found to be identical in different strains, masking huge variations in other genomic regions (Leconte et al., 2020).

To address the limitations of metabarcoding, metagenome read mapping and metagenome-assembled genomes (MAGs) have emerged as valuable tools for biogeographic studies. Mapping metagenomic reads to reference genomes provides a more accurate and sensitive method for biogeographic quantification, avoiding targeted amplicon biases and gene content variations, and enabling higher strain resolution. This approach has been successfully applied to six Mamiellales genomes from the well-studied genera (*Micromonas*, *Ostreococcus* and *Bathycoccus*) using global metagenomic data from the Tara Oceans Expedition (Leconte et al., 2020). However, the lack of reference genomes from cultured strains has made achieving genome-resolved metagenomic mapping with species resolution challenging for *Mantoniella*. Although a *Mantoniella* MAG was identified in one Tara metagenomic sample from the southwestern Pacific Ocean (Delmont et al., 2022), the binning of this MAG was based on marker genes, resulting in an incomplete MAG.

Existing research on the global biogeography of Mamiellophyceae has primarily focused on regions outside the western Pacific Ocean, particularly overlooking subtropical coastal South Asia. Localised studies conducted in the West Philippine Sea (Dela Peña et al., 2021), Bohai Sea (Xu et al., 2017), South China Sea (Wu et al., 2014; Lin et al., 2017a, 2021) and East China Sea (Lin et al., 2017b, 2022) have highlighted the importance of factors like distance to the coast and depth in shaping the distribution patterns of Mamiellophyceae species. Despite these efforts, limited information exists on the occurrence of the genus *Mantoniella* in subtropical West Pacific waters, with studies indicating its presence at certain sites but without detailed species identification (Lin et al., 2017b, 2021, 2022). Notably, no cultures of *Mantoniella* have been isolated from the Pacific Ocean. Globally, only a few large-scale metabarcoding studies have investigated the biogeography of *Mantoniella* (Lovejoy et al., 2007; Tragin and Vaulot, 2019; Yau et al., 2020b).

Our study addresses research gaps on the biogeography of Mamiellophyceae, focusing specifically on the genus *Mantoniella.* We present a newly identified species, *Mantoniella tinhauana*, isolated from the subtropical coastal waters of the South China Sea. This represents the first-ever whole genome project for *Mantoniella*. By analysing the genome of this novel species and exploring its global distribution using metagenome datasets, we provide insights into its morphology, phylogeny, genome characteristics, evolutionary relationships and ecological niches. Our findings contribute to the understanding of the ecological significance and biogeography of Mamiellophyceae, furthering our knowledge of these important photosynthetic organisms in marine ecosystems.

## Materials and methods

### Sampling and strain isolation

Coastal surface seawater samples (2 m depth) were collected from Lau Fau Shan in the Pearl River Estuary (22°28′09.0′N 113°58′50.1′E) in June 2020. The collected samples were sequentially filtered through 50 μm mesh and 1 μm polycarbonate filters (Whatman) to remove large multicellular organisms and capture the suitable size fraction for Mamiellophyceae (Yung et al., 2022). The filtrates were grown in flasks (SPL Life Sciences) in L1 medium (Guillard and Hargraves, 1993) and cultured at room temperature (21–23°C) in a shaking incubator (100 rpm) under a 12-h light/dark cycle (30 μmol photons m^−2^ s^−1^). After 2–3 weeks, collected algal pellets were used for 18S rRNA gene amplification (see below) to obtain initial taxonomic classification. Samples containing Mamiellophyceae were purified through serial dilution and treated with antibiotics. The novel *Mantoniella* strain obtained from this process was subcultured.

### Morphology

For transmission electron microscopy (TEM), culture was centrifuged, the pellet resuspended and fixed with glutaraldehyde (2.5% in 0.2 μm-filtered seawater), rinsed with Na cacodylate buffer (0.1 mol l^−1^) and fixed in OsO_4_ (1%), then dehydrated with a graded series of acetone solutions. After resin embedding and polymerisation, ultrathin sections were cut using a Leica EM UC7 Ultramicrotome and stained with uranyl acetate and lead citrate. The stained samples were examined under a Hitachi HT7700 Transmission Electron Microscope. Cell diameters were measured using the TEM images and scales as viewed on ImageJ (Collins, 2007).

### Nucleic acid extraction and sequencing

For phylogenetic analysis, DNA was extracted from dense *Mantoniella* culture by centrifugation, resuspension and incubation at 98°C for 10 min, based on Saulnier et al. (2009) and Jahn et al. (2014). The 18S region was amplified following the protocol described by Hadziavdic et al. (2014) and Sanger sequenced by Tech Dragon (Hong Kong).

For whole genome sequencing, DNA was extracted from *Mantoniella* pellets using a Qiagen DNeasy Plant Pro Kit. Short-read Illumina sequencing was conducted by Novogene (Hong Kong) on the Novaseq 6,000 PE150 platform. Long-read DNA was extracted using a modified CTAB protocol (Stark et al., 2020) and sequenced on the PacBio SMRT Sequel platform by Novogene. To improve assembly contiguity, genetic material was extracted for proximity ligation (Hi-C) based on the protocol by Lafontaine et al. (2021). The chromatin was cross-linked following the instructions provided by Phase Genomics (United States) and subsequently subjected to Illumina Hi-C sequencing.

To improve gene prediction, a time-series of samples was collected from an exponentially growing culture every 3 h over a 24-h period. RNA was extracted using a Direct-zol RNA Miniprep Kit (Zymo Research) to capture the gene activity throughout the day. The RNA from the 8 timepoints was pooled and sequenced using Illumina PE150 by Novogene.

### Whole genome assembly

Paired-end Illumina reads were trimmed with trimmomatic v0.39 (Bolger et al., 2014) and quality controlled with fastp v0.23.2 (Chen et al., 2018). The short Illumina reads and long PacBio reads were combined to generate an initial hybrid assembly using MaSuRCA v4.0.9 (Zimin et al., 2013). Subsequently, the assembly was refined using POLCA (Zimin and Salzberg, 2020) for calling alternatives and improving accuracy.

For contig scaffolding and misassembly correction, Hi-C reads were aligned to the draft genome assembly using bwa-mem2 v2.0 (Li and Durbin, 2009). The resulting alignments were input into SALSA2 v2.3 (with default parameters and “-e GATC --clean”) (Ghurye et al., 2019).

Anvi’o v7.1 (Eren et al., 2015) was used to create a contigs database for removing extraneous sequences in the draft genome. Contigs were split into 2000 nt lengths, clustered and binned. HMM modelling and functional annotation against NCBI’s COGs database (Tatusov et al., 1997) were performed. Annotated bins and GC contents were visually examined, and genome bins not belonging to eukaryotic nuclear DNA were manually removed (Delmont and Eren, 2016).

To further enhance the draft genome, the initial trimmed and quality-controlled Illumina reads were realigned to the decontaminated draft genome using Bowtie2 v2.2.5 (Langmead and Salzberg, 2012). The resulting alignment underwent gap fixing, misassembly identification, variant calling, and other improvements using Pilon v1.24 (Walker et al., 2014).

### Marker gene characterisation

Mamiellophyceae 18S rRNA genes and Mamiellaceae ITS region sequences were aligned onto the novel *Mantoniella tinhauana* genome assembly using Geneious Prime v2022.2.2. The resulting 18S gene sequence obtained from this mapping approach was 1,784 bp in length and showed 100% identity to the amplicon sequencing-derived sequence, with greater completeness (deposited to GenBank: OR835992). To extract the ITS2 gene, the novel *Mantoniella* ITS region (deposited to GenBank: OR835993) was submitted to the University of Würzburg ITS2 Database (Merget et al., 2012) online platform using the Viridiplantae model and default parameters, resulting in a 253 bp-long ITS2 gene sequence.

### ITS2 structure

The novel *Mantoniella* ITS2 structure was predicted using Vienna files without gaps from Yau et al. (2020b) as templates on the ITS2 Database (Merget et al., 2012). The resulting Vienna file was exported and aligned with other Mamiellophyceae ITS2 structure files (including gaps) using ClustalW v2.1 (Aiyar, 1999) in 4SALE v1.7.1 (Seibel et al., 2006) (Supplementary Table S1). The novel ITS2 structure was visualised using the ViennaRNA web service forna (Gendron et al., 2001). Each base pair in the helix structures was compared to those of other *Mantoniella* and Mamiellophyceae ITS2 using the 4SALE alignment and manually labelled. Individual RNA helix sequences were extracted using Geneious Prime and also drawn on the ViennaRNA forna platform.

### Marker gene-based phylogeny

To construct the phylogenetic tree based on the 18S marker gene, the *M. tinhauana* 18S rRNA gene sequence was blasted against the NCBI nucleotide database in Geneious Prime (Supplementary Table S2). The top 10 matches, along with other complete *Mantoniella* 18S sequences (Tragin and Vaulot, 2019; Yau et al., 2020b) and representatives of other Mamiellophyceae 18S sequences were used to create the phylogenetic tree.

A total of 41 Mamiellophyceae 18S sequences, including the novel strain, were aligned using MAFFT v7.453 (Katoh and Standley, 2013). Low-quality positions containing gaps in over 50% of the sequences were removed with Goalign clean sites v0.3.5 (Lemoine and Gascuel, 2021). Maximum likelihood (ML) trees were built using IQ-TREE v2.2.0 (Nguyen et al., 2015) using the LG + F + R4 model of substitution and generating Shimodaira-Hasegawa (SH)-like approximate likelihood ratio test (aLRT) branch support values from 1,000 replicates. Markov chain Monte Carlo iterations were performed on the alignments for 1,000,000 generations sampling every 100 generations with 100,000 burn-in length using MrBayes v3.2.6 (Ronquist et al., 2012) as implemented on Geneious Prime, generating Bayesian posterior probability values for each node. The resulting tree was visualised using Interactive Tree Of Life (iTOL) v5 (Letunic and Bork, 2021).

To construct the ITS2 marker gene-based phylogenetic trees, gapped ITS2 sequences from 15 Mamiellophyceae species were aligned. ML and Bayesian trees were generated using the same methods as above.

### Genome and proteome analysis

To assess the completeness of the draft genome and Mamiellophyceae reference genomes, BUSCO v5.4.3 (Simão et al., 2015) was run against the Chlorophyta universal single-copy marker database in “genome” mode. Scaffold ends were manually examined for telomeric tandem repeats and scaffold and whole genome GC contents were computed using Geneious Prime.

The ploidy of the novel Mantoniella genome was estimated by aligning Illumina reads against the draft genome using ploidyNGS v3.1.3 (Corrêa dos Santos et al., 2017).

Transposable elements in the draft genome were identified using RepeatModeler v2.0.4 (Flynn et al., 2020), followed by masking of these elements, interspersed repeats and low complexity DNA sequences using RepeatMasker v4.1.4 (Smit et al., 2015) with RMBlast v2.13.0. Transcript reads from the transcriptome time-series were trimmed with trimmomatic, quality controlled with fastp as before, and aligned to the RepeatMasker-masked genome using STAR v2.1.10 (Dobin et al., 2013). The masked genome and the RNA-seq STAR alignment were input into BRAKER v3.0.2 (Brůna et al., 2021) for protein coding gene structure prediction in the novel genome using GeneMark and AUGUSTUS (Lukashin and Borodovsky, 1998; Keller et al., 2011), following BRAKER pipeline B.

The transcript reads were ribodepleted with SortMeRNA v4.3 (Kopylova et al., 2012) and assembled into a transcriptome with rnaSPAdes v3.15.4 (Bushmanova et al., 2019). Assembly quality was assessed using BUSCO in “transcriptome” mode against the Chlorophyta database, resulting in 94% complete BUSCOs. Open reading frames (ORFs) within the transcriptome were identified with Transdecoder v5.7.0 (Haas, 2023).

The unmasked genome, *ab initio* predictions from GeneMark and AUGUSTUS, output from BRAKER, and transcriptome ORF predictions from Transdecoder were combined in EVidenceModeler v2.0.0 (Haas et al., 2008) to generate a consensus proteome and a gene predictions file. The whole novel proteome was functionally annotated using HMMER v3.3 (Eddy, 2011) to generate Pfam (Mistry et al., 2021) domain annotations.

### Gene synteny and collinearity

Mamiellophyceae proteomes (excluding *O. mediterraneus*) were obtained from NCBI. Gene synteny comparison was performed using the *M. tinhauana* gene predictions and proteome outputs from EVidenceModeler, along with those of the five other Mamiellophyceae species. The six proteomes were concatenated and subjected to all-against-all blastp analysis. MCScanX (Wang et al., 2012) was used to compare syntenic gene blocks with a minimum block size of five genes and a maximum gap size of 25 genes (Cho et al., 2023). The collinearity output was visualised using SynVisio in Multi-Level Analysis Tree View mode (Bandi et al., 2022).

The Big Outlier Chromosome (BOC), the mating type locus (MT) region within each BOC as delimited by Benites et al. (2021), and the Small Outlier Chromosome (SOC) annotated sequences were extracted from each Mamiellophyceae proteome. Gene block synteny analysis was performed using MCScanX as described above, comparing the BOC/MT/SOC regions of each Mamiellophyceae species against the entire *Mantoniella* proteome.

### Orthogroup and gene expansions and contractions analysis

Orthogroups in the six proteomes were compared using Orthofinder v2.5.4 (Emms and Kelly, 2019) and visualised using UpSetR (Conway et al., 2017). Orthogroup information and the species tree produced by Orthofinder (made ultrametric) were used for gene family expansions and contractions analysis in CAFE v5 (Mendes et al., 2021) with a *p*-value cutoff of 0.05. The expansion and contraction tree was drawn using cafe5_draw_tree.py and pie charts for each tree node were added using meta-chart.com. Significantly expanded families in *M. tinhauana* were functionally annotated using eggNOG-mapper v2.1.9 (Cantalapiedra et al., 2021) with DIAMOND (Buchfink et al., 2021) and the eggNOG v5.0 database (Huerta-Cepas et al., 2019). GO enrichment analysis was performed following the published protocol (Zhou, 2022) in R and plotted using ggplot2 (Wickham, 2006).

### Global distribution

Metagenomic reads from the Tara Oceans dataset were obtained from two projects (PRJEB4352-global and PRJEB9691-polar) through the European Nucleotide Archive in 2023. The samples were collected from the ocean surface at a depth of 5 m. The size fraction of interest was >0.8 μm (>0.8 μm, 0.8–5 μm or 0.8–20 μm samples were collected from different sites). In cases of site duplication, preference was given to >0.8 μm samples. A total of 124 metagenomic samples were mapped onto the full *M. tinhauana* genome and the *Micromonas pusilla* CCMP1545 genome (Worden et al., 2009) as a control using BBMap v38.96 (Bushnell, 2016) with a minimum identity of 95%. The mapping values were compiled using pileup.sh from the BBMap suite (Malfertheiner et al., 2022). To account for variations in total read numbers and genome lengths, Reads per Kilobase per Million (RPKM) values were computed as follows:

$$\begin{array}{l} \mathrm{RPKM}=\mathrm{mappedReads}/\\ \left(\mathrm{genomeLength}/1000^{*}\;\mathrm{totalReads}/1,000,000\right) \end{array}$$

The coordinates and RPKM were collated. Custom code in R, along with the ggplot2 (Wickham, 2006) and scatterpie (Yu and Yu, 2018) packages, were utilised to plot the data onto world maps. Median values for environmental measurements for each sample site were downloaded (Supplementary Table S3). Spearman’s rank correlation coefficients (SRCC) were calculated in R for each pair of environmental variables, and half the variables with more than 0.5 pairwise correlation were removed. The variables that were not highly collinear (only temperature, salinity and nitrate) were used for SRCC calculation with the *M. tinhauana* RPKM and plotted with ggpairs (GGally) (Schloerke et al., 2018). Some nitrate values were missing and so those samples were excluded from the nitrate-RPKM correlation study.

## Results and discussion

### Isolation, taxonomic classification and morphological characterisation of a novel *Mantoniella* species

In an effort to isolate *Mantoniella* species from coastal waters in the Western Pacific, a sweep of green algae from water samples were collected along the coast of Hong Kong in different seasons and subsequent purification using serial dilutions was performed. To assess their evolutionary distance compared to other cultured Mamiellophyceae, we sequenced and compared their partial 18S rRNA gene with algal sequences in GenBank. We discovered that one algal culture exhibited a remarkably high similarity to *Mantoniella squamata*, as evidenced by a 99.7% nucleotide identity.

**Figure 1:**
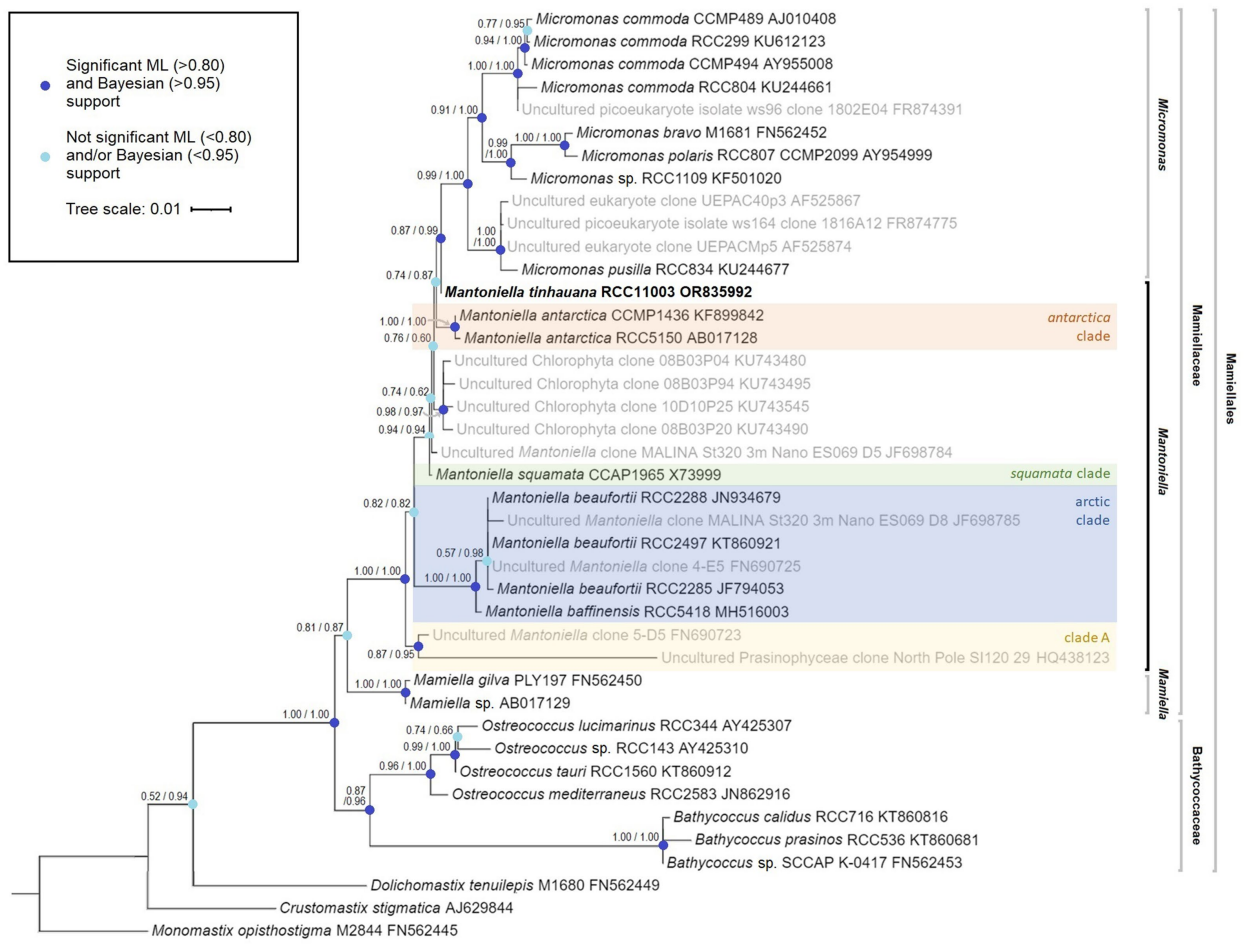
Mamiellophyceae 18S rRNA gene maximum likelihood tree, featuring species name, species code and 18S GenBank code (*Monomastix opisthostigma* as the outgroup). The numbers represent node support values, with the SH-like support values followed by the Bayesian posterior probability values. Nodes with enough support from both values (SH>0.80 and Bayes>0.95) are indicated in dark blue whereas nodes lacking sufficient support from either or both values are marked in light blue. Uncultured strains, obtained through blast search and previous studies, are depicted in grey where a complete 18S sequence was available. *Mantoniella* clades, if known, are labelled in different colours.

To determine the taxonomic classification of this newly isolated alga, we constructed a phylogenetic tree based on the complete 18S rRNA gene. This analysis positioned the alga on the outer boundary of the *Mantoniella* genus, showing a close affinity to *Micromonas* (Figure 1). We named this novel species *Mantoniella tinhauana*. To aid in classification, *Mantoniella* strains were assigned to clades based on this phylogeny and previous studies (Tragin and Vaulot, 2019; Yau et al., 2020b). The novel strain had a very high shared identity (up to 99.9%) with several partial 18S sequences, for example strains assigned to clade B for which only the 18S V4 region was available (Tragin and Vaulot, 2019). As complete 18S sequences were lacking for certain strains (these were excluded), the novel *Mantoniella* could not be assigned to any known clade based on the whole 18S gene. As discussed before, relying solely on the 18S or any single marker gene for phylogenetic assessment may not be entirely reliable, especially in unicellular eukaryotes. This limitation is particularly evident in a genus like *Mantoniella*, which only has a few cultured and extensively studied species. To address this, we also examined the ITS2 marker.

**Figure 2:**
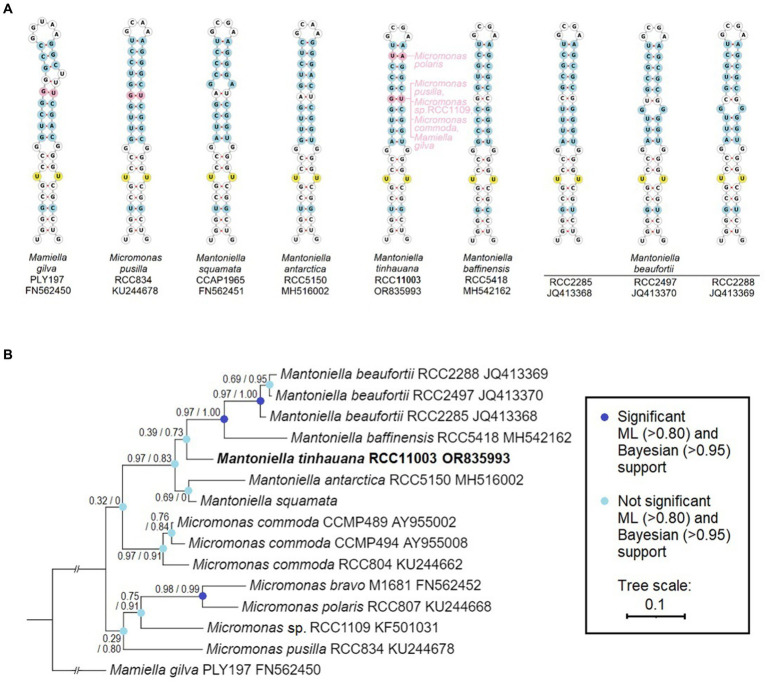
ITS2 RNA helix structural and base comparison, and phylogeny of Mamiellaceae. **(A)** ViennaRNA forna ITS2 folding structure of helix 2 (the other helices and complete ITS2 structure can be found in Supplementary Figures S1, S2), base-by-base comparison of novel *Mantoniella tinhauana* to other *Mantoniella* and Mamiellaceae strains, displayed in ITS2-based phylogenetic order. Only one representative *Micromonas* species and one *Mamiella* are shown. Yellow: universal eukaryote ITS2 motifs. Blue: site of nucleotide variant present in other *Mantoniella* species with structural effect. Pink: site of nucleotide variant absent in other *Mantoniella* species but present in other Mamiellophyceae species. **(B)** Mamiellaceae phylogenetic tree based on the structurally-informed comparison of ITS2 sequences, with branch support values as described.

The analysis of the ITS2 sequences in *M. tinhauana* revealed the presence of eukaryotic universal hallmark motifs (Mai and Coleman, 1997; Schultz et al., 2005), namely a Y-Y (U–U) mismatch in helix 2 and a YRRY (UGGU) motif in helix 3. These motifs were consistently identified in all ITS2 sequences (highlighted in yellow in Figure 2A and Supplementary Figures S1, S2). We further examined the folding patterns of their ITS2 molecules to differentiate between base differences that have structural effects and compensatory base changes shared with related Mamiellophyceae species. In helix 2 (Figure 2A), we observed that approximately half of the bases were identical in all Mamiellophyceae (white, yellow), while the other half exhibited variations that were also present in other *Mantoniella* species (blue). Additionally, two base pairs were exclusive to *M. tinhauana* and *Micromonas* or *Mamiella* species (pink). Similar differences were observed in the other three helices (Supplementary Figures S1, S2). Based on the ITS2 helix folding structures, *M. tinhauana* shares a mix of base pair variants characteristic of arctic *Mantoniella* species and *M. squamata*, placing it firmly within the *Mantoniella* genus. Interestingly, it also possesses some structural base pairs that are unique to *Micromonas* or *Mamiella* strains.

In addition to visualising and comparing the structural differences in ITS2, we constructed a phylogenetic tree based on the structurally-informed ITS2 alignments (Figure 2B). This ITS2 tree differs significantly from the phylogeny based on the 18S gene, although branch support values are predominantly low. The discrepancies between the ITS2 and 18S trees can be attributed to varying evolutionary rates and histories, hybridisation events, and selection pressures (Baldwin et al., 1995; Hershkovitz and Lewis, 1996).

**Figure 3:**
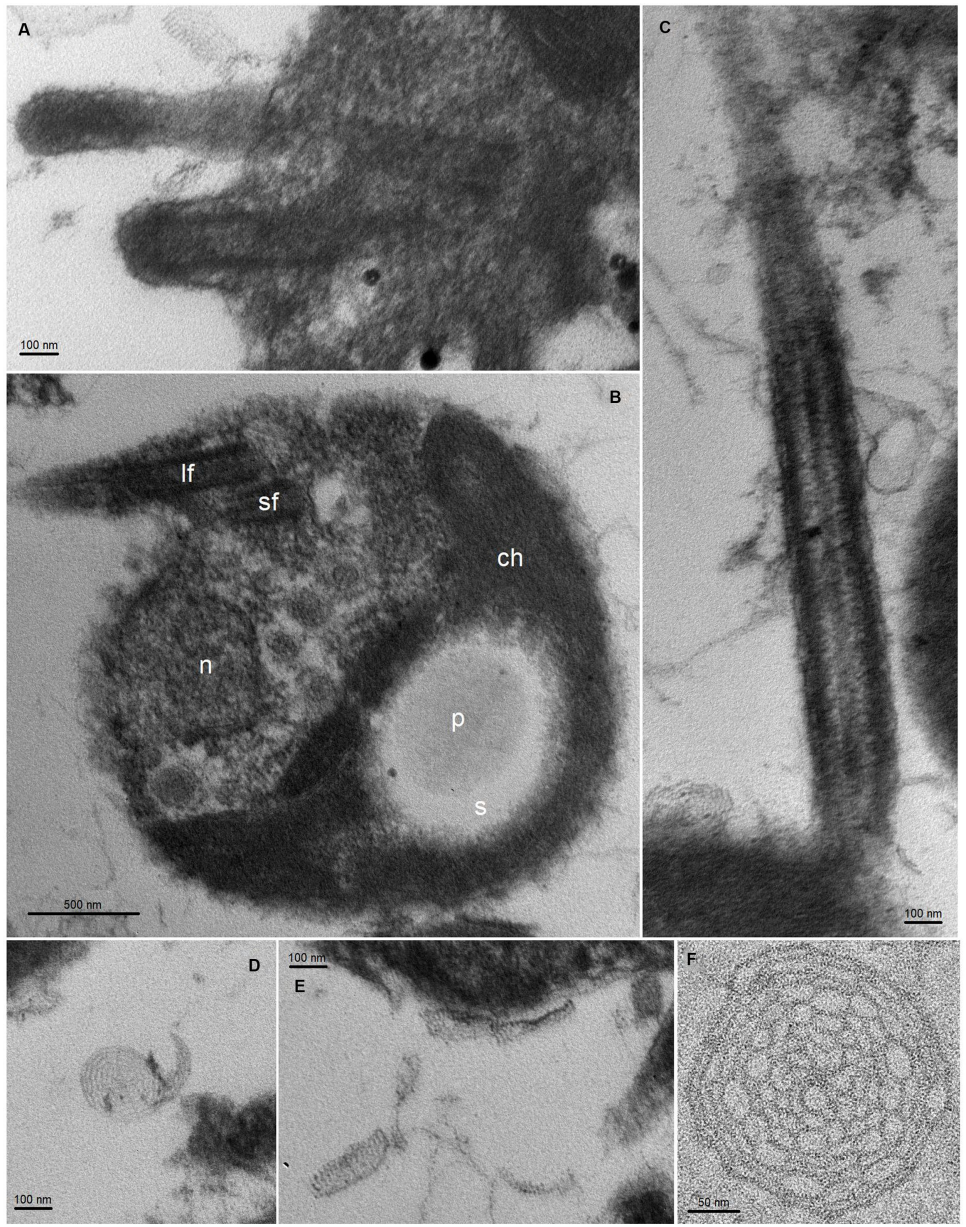
TEM thin sections of *Mantoniella tinhauana*. Cells were measured to be approximately 3 μm in diameter (10 cells measured, standard deviation 0.7 μm, min size 1.8 μm, max size 4.5 μm, median 3.1 μm), slightly smaller than but around the range of *M. squamata* (3–6.5 μm) (Manton and Parke, 1960), *M. antarctica* (2.8–5 μm) (Marchant et al., 1989), *M. beaufortii* (2.9–5 μm) and *M. baffinensis* (3.5–5.7 μm) (Yau et al., 2020b). **(A)** Bases of the long and short flagella. **(B)** Whole cell view with detail of organellar structures: n = nucleus, ch = chloroplast, s = starch granule, p = pyrenoid, lf = long flagellum, sf = short flagellum. **(C)** Detail of flagellum covered in scales (body and hair scales). **(D–F)** Body scales with octaradial spiderweb structure.

We conducted transmission electron microscopy (TEM) analysis of *M. tinhauana,* revealing cells larger than typical *Ostreococcus, Bathycoccus* and *Micromonas* cells – with cell sizes increasing, respectively, from 0.6 to 3 μm, as reported in previous research (Manton and Parke, 1960; Eikrem and Throndsen, 1990; Chrétiennot-Dinet et al., 1995; Kuroiwa et al., 2004). The cells of *M. tinhauana* measured between 1.8 and 4.5 μm, with characteristic features commonly observed in cells of the *Mantoniella* genus (Figure 3). No features unique to the new species *Mantoniella tinhauana* were identified, similar to previous observations of *M. baffinensis* and *M. beaufortii* (Yau et al., 2020b). These species exhibited slight differences in size but overall resembled *M. squamata*. One exception was the radiating pattern on the spiderweb scales, which allowed differentiation of *M. beaufortii* from the other two species based on the number of radial spokes. *M. tinhauana* exhibited spiderweb-like octaradial scale symmetry just like *M. squamata* and *M. baffinensis*. In any case, morphological differences among *Mantoniella* species are minor, prompting us to investigate genetic differences as the next step in our research.

### *Mantoniella tinhauana* exhibits double the genome size of other Mamiellophyceae species

The first-ever *Mantoniella* strain whole genome was generated using short read paired-end sequencing, PacBio SMRT sequencing, and Hi-C sequencing techniques. The completeness of the assembly was assessed using BUSCO analysis against the Chlorophyta database, which indicated a high completeness value of 97.1% (Supplementary Table S4), comparable to other Mamiellophyceae reference genomes (Table 1). The *de novo* assembly yielded a 40 Mb genome composed of 27 scaffolds, with longer scaffolds compared to other Mamiellophyceae species (Supplementary Table S5a). However, only a low percentage of *M. tinhauana* scaffolds contained telomeres. Specifically, 11 scaffolds had telomeric tandem repeats on one end [CCCTAAA or the reverse complement TTTAGGG, the same as other Mamiellophyceae and plants (Richards and Ausubel, 1988)], and only four scaffolds had telomeres on both ends. This indicates imperfect genome assembly, a common issue observed in other reference genomes such as *B. prasinos* (Table 1). Despite the larger genome size and increased number of scaffolds, ploidy prediction confirmed that *M. tinhauana* is haploid, consistent with other known Mamiellophyceae species. Repeat masking revealed that 14.9% of the genome was composed of repeat elements (Supplementary Table S6), a significantly higher proportion (double) than seen in other Mamiellophyceae (Xu et al., 2022).

**Table 1:** Mamiellales genomes used in this study and their characteristics.

Species	Genome size (Mb)	Chromo-some /scaffold number	Genome GC (%)	% scaffold ends with telomeres	% scaffolds with two telomeres	BUSCO completeness (%)
*Ostreococcus tauri* RCC4221 (Blanc-Mathieu et al., 2014)	12.9	20	59.4	67.5	35.0	98.1
*Ostreococcus lucimarinus* CCE9901 (Palenik et al., 2007)	13.2	21	60.4	100	100	98.8
*Ostreococcus mediterraneus* RCC2590 (Yau et al., 2020a)	13.9	20	56.2	60.0	40.0	97.8
*Bathycoccus prasinos* RCC1105 (Moreau et al., 2012)	15.0	19	48.1	34.2	10.5	97.1
*Micromonas commoda* RCC299 (Worden et al., 2009)	21.0	17	64.0	100	100	99.1
*Micromonas pusilla* CCMP1545 (Worden et al., 2009)	22.0	21	65.9	90.5	90.5	98.0
*Mantoniella tinhauana* RCC11003	39.5	27	64.8	35.2	14.8	97.1

Except for *M. tinhauana*, marked in green, all genomes and the size and chromosome/scaffold information were obtained from NCBI.

*Mantoniella tinhauana* represents the species with the largest recorded genome size within the Mamiellophyceae class to date. This species also exhibits larger cell sizes compared to other Mamiellales species, prompting the question of a possible correlation between genome size and cell size within the Mamiellophyceae class. Indeed, *Ostreococcus* species have the smallest cells (Chrétiennot-Dinet et al., 1995) and correspondingly small genomes. This trend continues with *Bathycoccus* species exhibiting slightly larger cells and genomes (Eikrem and Throndsen, 1990), and *Micromonas* following with even larger dimensions (Manton and Parke, 1960), as shown in Table 1. However, the number of scaffolds or chromosomes does not appear to follow a similar trend. A study contrasting the cell and genome sizes of *O. tauri* and a red algal species suggested that cell and genome sizes are not interdependent (Kuroiwa et al., 2004), but the comparison involved two phylogenetically distant species, potentially obscuring any correlation due to vastly different genomic structures and contents. To date, a systematic comparison of cell and genome sizes within Mamiellophyceae has not been undertaken. Preliminary observations hint at a significant correlation within this group. To test this hypothesis, further genomic sequencing and assembly of larger-celled Mamiellophyceae species, particularly from the genera *Mantoniella* or *Mamiella* (Alonso-González et al., 2014), would help validate this relationship between genome and cell sizes in this phylogenetic group.

### Gene block synteny and chromosomal rearrangements

**Figure 4:**
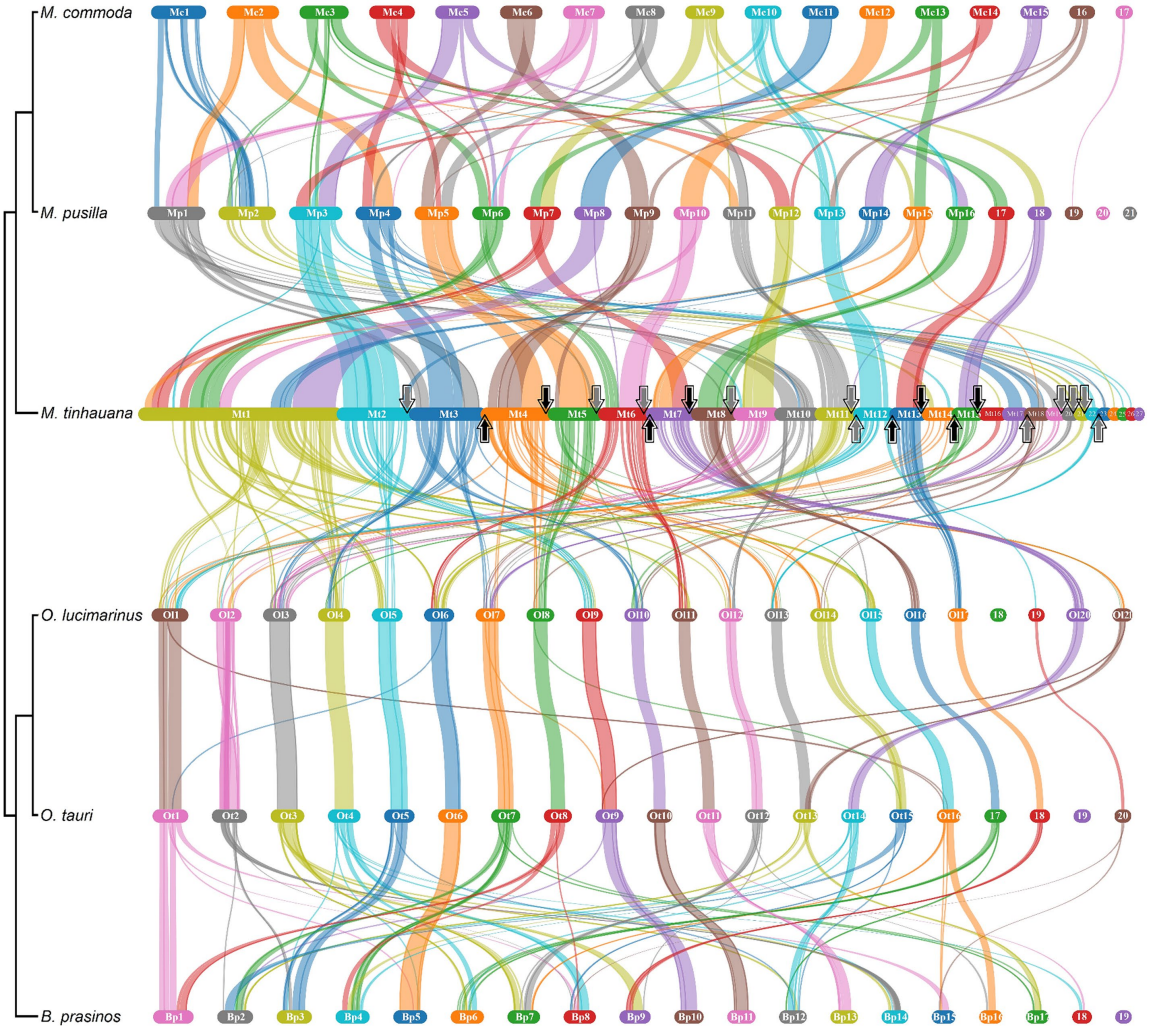
Pairwise gene block synteny of Mamiellales proteomes computed with MCScanX and visualised using SynVisio. Species are ordered by 18S-based phylogeny, with a simplified phylogenetic tree and names labelled on the left. Scaffolds or chromosomes for each species are shown in separate sections of different colours (random repeating 10 colour series), in order (largest to smallest), labelled. Collinear gene blocks between each pair of species are connected with ribbons of varying thicknesses denoting gene block lengths, in the colour corresponding to the source scaffold. For *M. tinhauana*, telomeres at the beginning (upwards arrow) or end (downwards arrow) of a scaffold are marked with arrows. The four scaffolds which have telomeres at both ends have black arrows.

Pairwise gene block synteny among the different Mamiellophyceae species is depicted in Figure 4. Notably, *M. tinhauana* exhibits similar grouping and colocalisation patterns of gene blocks as those of *Micromonas* species, but with longer gene blocks, indicating gene block duplications and expansions. *O. lucimarinus* and *O. tauri* have nearly identical chromosome and gene block order. In contrast, *Micromonas commoda* and *Micromonas pusilla*, despite belonging to the same genus, display more frequent chromosomal order and gene block rearrangements, similar to those observed between *Micromonas pusilla* and *M. tinhauana*. These findings suggest that there have been more rearrangements and less consistent arrangement of syntenic blocks in the Mamiellaceae compared to the Bathycoccaceae, and *M. tinhauana* follows this trend. Bathycoccaceae chromosomes tend to be shorter, resulting in fewer gene blocks corresponding to different chromosomes, whereas the Mamiellaceae have longer chromosomes, increasing the likelihood of rearrangements. Given that *M. tinhauana* possesses the longest chromosomes, it is unsurprising that gene blocks from multiple chromosomes in other species correspond to syntenic blocks on a single chromosome in *M. tinhauana*, and vice versa. Importantly, 75.7% of genes across all species exhibited collinearity, which suggests that, despite the rearrangement of gene blocks among different species, there is a substantial conservation of both gene content and, to a certain extent, the order of these blocks are correctly assembled in the novel species genome. This degree of collinearity provides substantial support to the accuracy of our genome assembly. Additionally, most *M. tinhauana* scaffolds with telomeres, particularly those with telomeres at both ends, demonstrate a higher level of synteny with other Mamiellophyceae species.

### Orthofinder analysis reveals species-specific characteristics of the novel *Mantoniella* proteome

**Figure 5:**
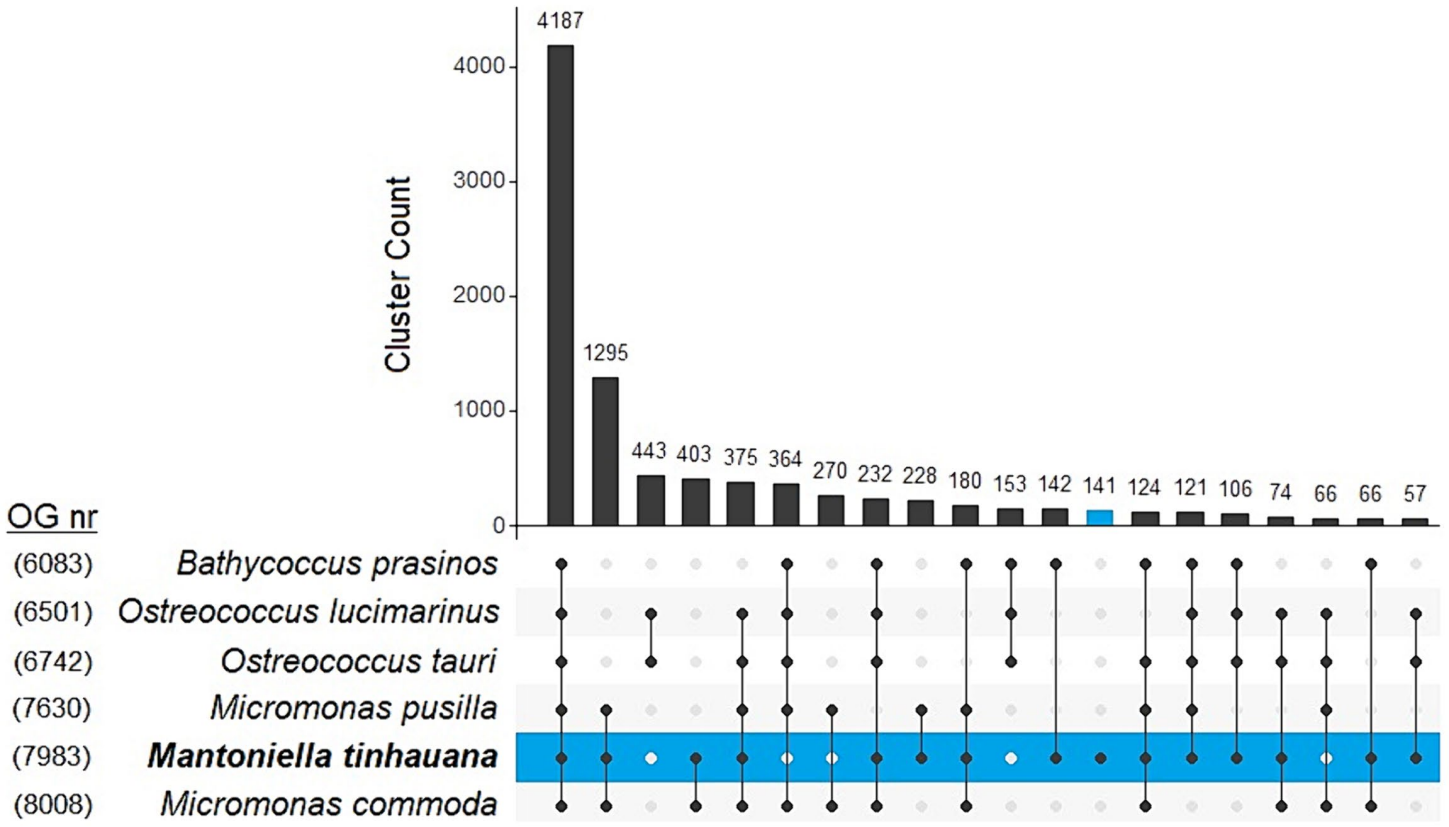
UpSet plot comparison of top 20 clustering unique and shared orthogroups in 6 Mamiellophyceae species. Number of orthogroups per species is labelled on the left.

In order to uncover the factors contributing to the larger genome of *M. tinhauana*, we conducted a comprehensive analysis of species orthogroups using Orthofinder. The overall and species-specific results of Orthofinder analysis can be found in Supplementary Table S7, with a graphical representation in Figure 5. The novel *Mantoniella* proteome exhibited notable characteristics, including the highest level of unassigned genes (1,288 or 11.6%), species-specific orthogroups (141 or 1.8%) and genes in species-specific orthogroups (394 or 3.5%). These findings were expected, considering that *M. tinhauana* is the first proteome in its genus and a newly discovered species with a significantly larger genome. The functional annotation of genes in *Mantoniella tinhauana*-specific orthogroups (188) is listed in Supplementary Table S8, which encompass a variety of roles, including motor proteins, carbohydrate metabolism and chromosome associated proteins. We further explored these species-specific orthogroups through Gene Ontology (GO) Enrichment Analysis.

### Functional significance of gene family expansions in *Mantoniella tinhauana*

**Figure 6:**
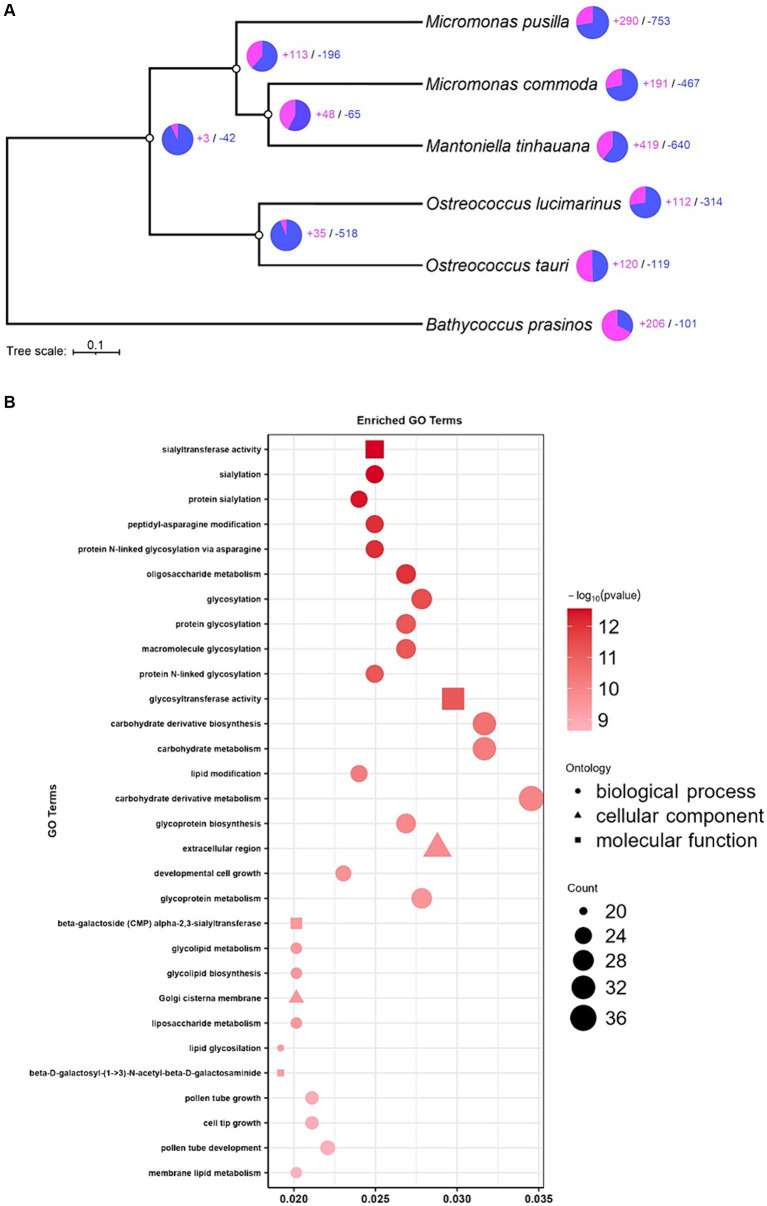
Expansion and contraction analysis and enrichment analysis. **(A)** Tree of expanded (purple) and contracted (blue) gene families. **(B)** GO enrichment analysis of significantly expanded gene families in *M. tinhauana*.

To gain a comprehensive understanding of the orthogroup differences between species, particularly those with assignable functions, we performed expansions and contractions analysis. This analysis sought to elucidate the evolutionary history and relationship of *M. tinhauana* and its close relatives. Interestingly, *M. tinhauana* exhibited numerous gene family expansions, and even more contractions (Figure 6A). Additionally, GO enrichment analysis (Figure 6B) provided insights into the biological functions associated with the most significant expansions.

GO enrichment analysis revealed strong expansions in glycosylation gene families, particularly sialylation, which may be involved in the development of *M. tinhauana*’s characteristic scales and will be discussed below. It also unveiled significant expansions in Golgi associated genes, which is where sialylation takes place in the cell (Schauer and Kamerling, 2018). Expanded gene families associated with carbohydrate metabolism and cell growth (Raven and Beardall, 2003; Mathieu-Rivet et al., 2020) suggest that *M. tinhauana* is a fast-growing species well-adapted to eutrophic waters, where it was isolated.

### Absence of lower GC content regions in *Mantoniella tinhauana*: implications for sexual reproduction, viral resistance and Mamiellophyceae speciation

Previously sequenced genomes of Mamiellophyceae in the genera *Micromonas, Ostreococcus* and *Bathycoccus* have a relatively high overall GC content and two lower GC content outlier chromosomes (Derelle et al., 2006; Piganeau et al., 2011b; Grimsley et al., 2015). These outlier chromosomes contain a higher proportion of transposable elements, leading to faster gene evolution and the presence of more species-specific, non-orthologous genes (Jancek et al., 2008). The BOC has distinct blocks with heterogeneous GC content, characterised by a section of lower GC flanked by normal, higher GC content. The low GC region suppresses recombination and contains one of two alternate mating type loci, which is hypothesised to predate Mamiellophyceae speciation (Blanc-Mathieu et al., 2017). In contrast, the SOC has low GC content throughout, and shows high variability in gene content even within the same species due to frequent duplications and internal rearrangements (Grimsley et al., 2015; Blanc-Mathieu et al., 2017). The SOC is associated with viral resistance (Moreau et al., 2012), as species possessing this variable chromosome demonstrate high viral sensitivity and the ability to rapidly develop resistance to viruses. SOC length is inversely correlated with viral susceptibility (Blanc-Mathieu et al., 2017). In unpublished experiments mentioned by Moreau et al. (2012), it was observed that two *Mamiella* and *Mantoniella* species do not exhibit the high viral sensitivity seen in the seven whole-genome sequenced species. These findings support the hypothesis that these species and potentially the entire genera may lack the SOC, although this has yet to be confirmed.

In the current study of the novel *Mantoniella* genome, no chromosomes with significantly lower GC content were identified (Supplementary Table S5b). Lower GC content is typically associated with suppressed recombination, which is a characteristic of sexual reproduction. However, no such regions were found in *M. tinhauana*, and direct observation of the process is virtually impossible. To confirm whether *M. tinhauana* undergoes sexual reproduction, we examined the presence of core meiosis genes (Derelle et al., 2006; Worden et al., 2009; Joli et al., 2017; Li et al., 2020), including the RWP-RK family of transcription factors known to be involved in gametogenesis in algae (Chardin et al., 2014). The findings demonstrate key meiosis genes are present at the expected levels in *M. tinhauana* (Supplementary Table S9), providing robust evidence supporting the existence of a sexual stage in this species.

In a study by Benites et al. (2021), the mean GC contents of coding sequences in Mamiellophyceae transcriptomes were compared to those of mating gene family coding sequences. Interestingly, some transcriptomes, including two *Mantoniella* transcriptomes, did not exhibit lower GC content in their mating gene family genes. In fact, certain species such as *Dolichomastix* even have a higher GC content in their mating genes. These findings support the possibility that earlier branching Mamiellophyceae gametologs are not confined to regions of lower GC content. It is conceivable that the genus *Mantoniella*, or at least *M. tinhauana*, never evolved to have recombination suppression in their mating gene regions, unlike *Bathycoccus, Ostreococcus* and *Micromonas* species. This could be because their mating genes are not clustered together on a single chromosome.

**Figure 7:**
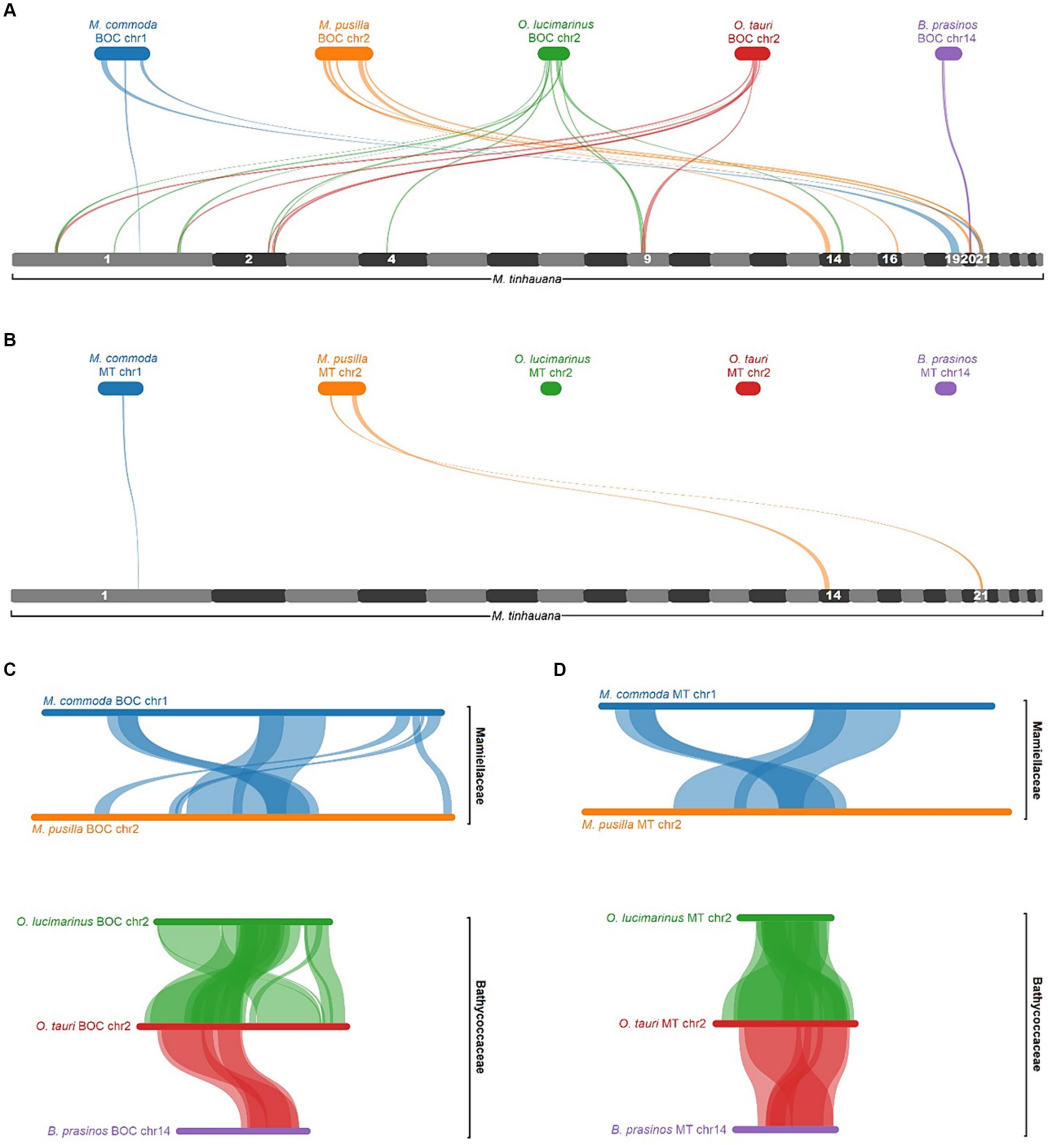
MCScanX pairwise alignment of BOCs and MTs. **(A)** BOC alignments to the full *Mantoniella tinhauana* genome. *M. tinhauana* scaffolds with syntenic blocks are numbered. **(B)** MT alignments to the *Mantoniella tinhauana* genome. **(C)** BOC alignments to one another. **(D)** MT alignments to one another. Collinear gene blocks (of at least 5 genes with a max gap of 25) are connected with ribbons of different thicknesses denoting gene block lengths, in the same colour as the source species.

To investigate further, we analysed the synteny of five Mamiellophyceae BOCs in comparison to the novel *Mantoniella* scaffolds. The gene blocks did not align with a single scaffold, but with several (Figure 7A). Notably, *M. tinhauana* shares syntenic blocks with all five species BOCs, which is not the case when comparing other Mamiellaceae to Bathycoccaceae BOCs (Figure 7C). This suggests that *M. tinhauana* has gene blocks in common with both groups, further supporting its phylogenetic placement between the two. However, these syntenic blocks are located on separate scaffolds, indicating a distinct genomic organisation. The BOC has previously been identified as emerging before the speciation of Mamiellophyceae (Blanc-Mathieu et al., 2017), and thus it should be present in all Mamiellophyceae species. The absence of the BOC in the novel *Mantoniella* genome could indicate three possibilities: (a) translocation or rearrangement after the branching of the *Mantoniella* genus, (b) a more recent evolutionary origin of the BOC than previously thought, or (c) assembly errors.

To determine if the syntenic BOC blocks were associated with mating, we examined gene block synteny between the MT loci within each BOC and the *Mantoniella* proteome (Figure 7B). The analysis revealed that only a few *Micromonas* MT gene blocks were found in *M. tinhauana*, proving that the majority of syntenic blocks between Mamiellophyceae BOCs and *M. tinhauana* are unrelated to mating. When comparing BOC gene blocks among Mamiellophyceae, the syntenic blocks primarily correspond to the MT loci (Figure 7D). These findings suggest that in *M. tinhauana*, the mating loci are not clustered and have undergone rearrangements and/or genetic divergence compared to its phylogenetic relatives.

Attempts to align the distinct and variable sequences of SOC in Mamiellophyceae with the *Mantoniella* genome were unsuccessful. Consequently, the SOC can only be identified based on chromosome length and GC content. Moreau et al. (2012) proposed that *Mantoniella* may lack a SOC due to its purported lower sensitivity to viruses. The absence of the SOC in this study supports this hypothesis, although SOC-lacking species could very well interact with viruses in different ways. We are currently studying the viral susceptibility of *M. tinhauana*. Further investigations should also aim to identify virus resistance genes present in other Mamiellophyceae and determine whether the same or orthologous genes are present in the *M. tinhauana* genome, with special attention to their location if present.

### Expanding gene families and scale development in *Mantoniella*

The gene family expansion analysis uncovered a significant expansion in sialylation and other glycosylation gene families in *M. tinhauana*. To investigate these expansions further, we conducted a targeted analysis on four protein families previously found to be expanded in *Bathycoccus prasinos* hypothesised to be involved in scale formation (Moreau et al., 2012). We searched for these families in the Pfam-annotated proteome of *M. tinhauana* and found similar copy number expansions in two out of the four families: glycosyltransferase family 29 and neuraminidase/sialidase (Supplementary Table S10). Another study by van Baren et al. (2016) also identified expansions in only two out of the four families in both scaled *B. prasinos* and *D. tenuilepis* compared to non-scaled species. Our findings confirm theirs, supporting the hypothesis that glycosyltransferase family 29 and sialidases are involved in scale development.

### Genetic basis of biflagellate phenotype in *Mantoniella*

The novel and other *Mantoniella* species possess two flagella, unlike other Mamiellophyceae genera that have one flagellum (*Micromonas*) or none (*Bathycoccus, Ostreococcus*). This difference in flagellar number should be reflected in their genomic characteristics, but gene family expansion analysis indicated no motor associated expansions. We analysed the Pfam-annotated proteome using a list of published flagella-related genes (Li et al., 2020). Comparison to other Mamiellophyceae revealed that *M. tinhauana* had higher copy numbers of certain microtubule synthesis and assembly protein domains compared to other species (Supplementary Table S11). A few cilia-, flagella- and motility-associated domains also showed a modest expansion in *M. tinhauana* compared to monoflagellated *Micromonas*. Marin and Melkonian (2010) proposed that the last common ancestor of the Mamiellophyceae had two flagella, as monoflagellate *Monomastix* and *Micromonas* species retain two basal bodies despite having only one flagellum. The identified gene expansions could potentially explain how *M. tinhauana* retained two flagella, but further functional studies are necessary to identify the genes involved in flagellar number and length control, as well as environmentally-stimulated and synchronous flagellar beating. These areas remain largely unexplored in Mamiellophyceae. Examining the expression of key genes, and not only their copy number, would provide further insights.

### Metagenomic analysis reveals the global distribution and environmental adaptation of *Mantoniella tinhauana*

**Figure 8:**
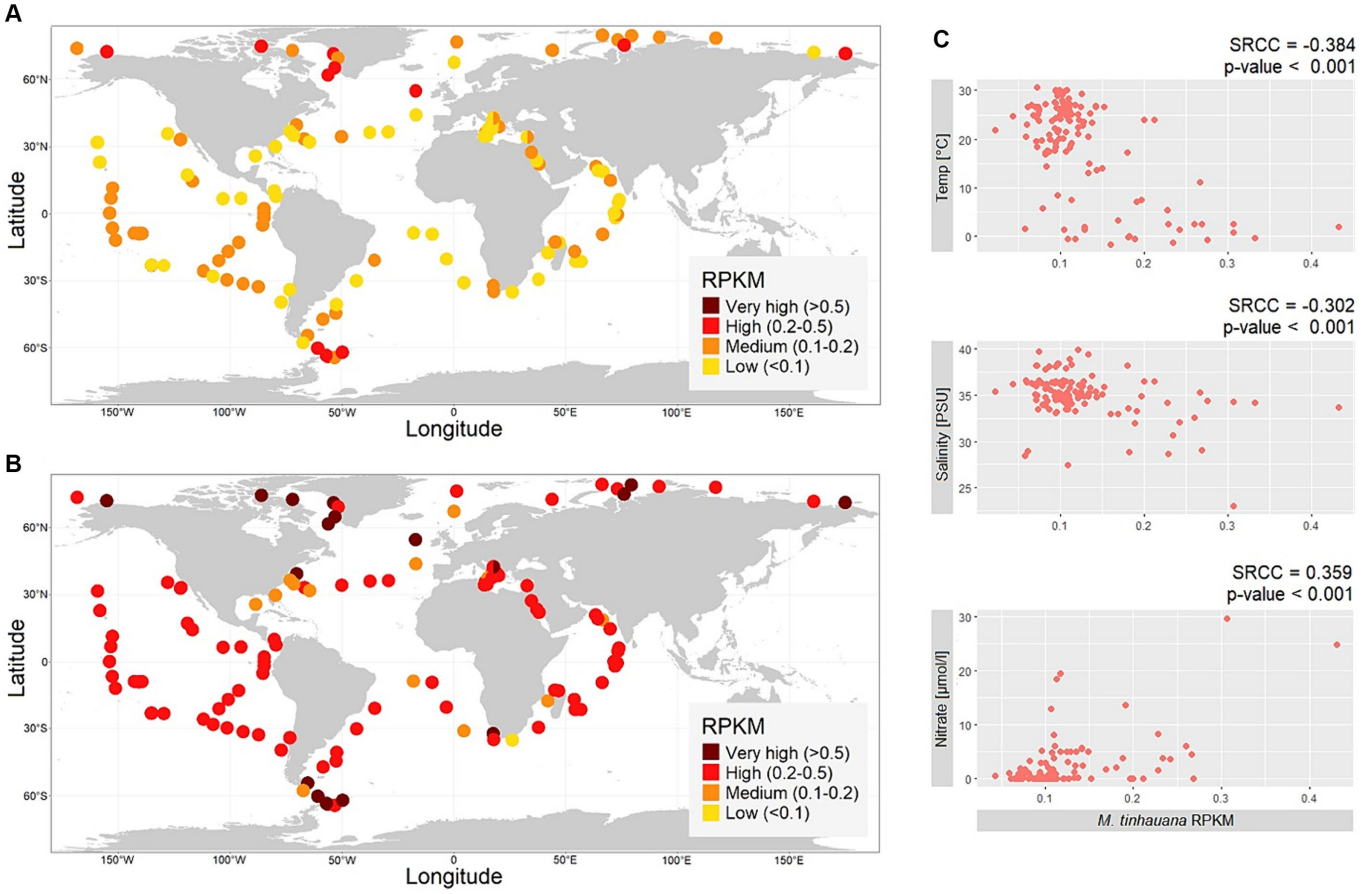
Biogeographic distribution of Mamiellophyceae. **(A,B)** Maps of global metagenomic RPKM read mapping of over 0.8 μm size fraction reads sampled at the ocean surface to **(A)** *Mantoniella tinhauana* genome, **(B)** *Micromonas pusilla* CCMP1545 genome. **(C)** Correlations between not highly collinear environmental variables and *M. tinhauana* RPKM values.

Metagenomic reads obtained from the Tara Oceans Expeditions were used to investigate the presence and abundance of *M. tinhauana*. Considering the size range of *M. tinhauana* cells (>1.8 μm) and *Micromonas pusilla* cells (>1 μm) (Manton and Parke, 1960), reads from the over 0.8 μm size fraction were used. These reads were mapped onto the novel and control genomes, and the relative abundance (RPKM) was calculated (Figure 8 and Supplementary Table S3).

Our analysis revealed the widespread occurrence of *Mantoniella tinhauana* reads across the tested sites in the surface of the global ocean. The abundance of *M. tinhauana* reads was consistently low, indicating that it is a cosmopolitan species with a low-level prevalence, except for polar regions where it was higher. The mean worldwide RPKM value of the tested sites was 0.12, with a maximum of 0.43 RPKM (1.73% of reads). The mapping to the control *Micromonas pusilla* CCMP1545 genome (global mean 0.36 RPKM, maximum 1.54 RPKM or 3.38% of reads) showed similar levels as reported previously (Leconte et al., 2020) using similar methods.

To understand the environmental factors influencing the abundance of *M. tinhauana*, we examined the correlation between read abundance and various environmental variables (Figure 8C). Although statistically significant, the individual relationships were not strongly pronounced, with the most correlated factor being temperature. Nitrate showed a positive correlation with RPKM, while temperature and salinity exhibited negative correlations. These findings support the hypothesis that *M. tinhauana* is adapted to growing in diverse nutrient levels, and in environments rich in nutrients, for instance with high nitrate, it can readily thrive and proliferate. The negative correlation between the abundance of *M. tinhauana* and temperature, and its higher presence in polar regions suggest a preference for cooler habitats within this genus. All species within the genus, with the exception of *M. squamata* and *M. tinhauana*, have been isolated exclusively from polar sites. This intriguing observation presents an exciting opportunity to conduct genomic comparisons between the non-polar and polar species, with the aim of unravelling the underlying genomic traits that contribute to their distinct adaptations to cold and higher temperatures, respectively.

## Taxonomy

### *Mantoniella tinhauana* RCC11003 Rey Redondo, Xu and Yung sp. nov.

#### Description

Illustrated in Figure 3. Round cells measuring 3 μm in diameter (standard deviation 0.7 μm, min size 1.8 μm, max 4.5 μm, median 3.1 μm), slightly smaller but around the range of other *Mantoniella* strains (Yau et al., 2020b). Biflagellate, with one long and one short flagellum. Cell body covered in octaradial spiderweb scales. One large chloroplast, occupying half the cell, with pyrenoid within starch granule. 18S and ITS2 rRNA sequences are species-specific (18S and ITS region GenBank accessions: OR835992 and OR835993, respectively).

#### Holotype

Isolated on the 11th June 2020 (10:30 am) from surface water (2 m depth) that was 30.5°C via peristaltic pump at the Lau Fau Shan coast of Hong Kong, in the Pacific Ocean (22°28′09.0′N 113°58′50.1′E). Culture deposited in The Roscoff Culture Collection under accession RCC11003.

#### Etymology

Tin Hau is the Cantonese name for the goddess of the sea revered in Hong Kong.

#### Habitat and ecology

Cosmopolitan species in surface water, with higher dominance in polar regions. Growth positively correlated with nitrate level and negatively correlated with temperature and salinity.

## Conclusion

We have discovered a novel species of Mamiellophycean green alga, *Mantoniella tinhauana*, from coastal surface water in the Western Pacific. Morphologically, it closely resembles other *Mantoniella* species, except for its smaller cell size. Through comparison of its 18S rRNA sequence and ITS2 structure with other Mamiellophyceae, we confirmed it to be a distinct species. Phylogenetically, it falls between the genus *Micromonas* and other *Mantoniella* strains.

The genome of *M. tinhauana*, the first draft genome of a *Mantoniella* species, was assembled with high completeness and annotated. It differs significantly from other Mamiellophyceae species, with a larger genome size (concurrent with larger cell size), more repeat elements and numerous unique genes. The larger genome size of the novel strain cannot be solely explained by the observed expansions. Comparative analysis with other Mamiellophyceae genomes revealed gene block duplications, expansions, as well as regions of no synteny and known function. To gain further insights, RNAseq should be conducted to determine the functions of more actively expressed genes.

Notably, *M. tinhauana* lacks the two low-GC outlier chromosomes found in all other studied Mamiellales, which are linked to mating and viral resistance. Despite the absence of synteny with BOC and MT loci seen in other species, the presence of meiosis genes in *M. tinhauana* suggests the existence of similar mating types and sexual reproduction mechanisms. These findings pose intriguing questions regarding the ancestry and evolution of Mamiellophyceae, which can only be hypothesised with a single divergent genome. Ongoing research on the viral infection dynamics of *M. tinhauana* may shed light on the absence of SOC and viral sensitivity in this algal class.

The analysis of expanded gene families revealed high glycosylation activity involved in scale formation. This and the two flagella in *Mantoniella* prompted a comparison of targeted gene groups between the novel species and other proteomes. While some investigated genes partially explained these structural differences, further study is required to fully understand the underlying mechanisms of scale patterns, flagella production and synchronised movement. The availability of this first whole genome of a biflagellate scaled Mamiellophycean species will undoubtedly contribute to future studies in these areas and beyond.

Future efforts should be focused on assembling and analysing the whole genomes of additional Mamiellophyceae species, including other *Mantoniella* strains and members of the understudied genus *Mamiella*. The challenges encountered in assembling the divergent *de novo* genome of *M. tinhauana* highlight the need for more cultures and complete genomes to enhance assembly accuracy and efficiency. Additionally, it would be valuable to assemble the plastid (chloroplast and mitochondrial) sequences of *M. tinhauana* and compare them to those of related species.

This study represents the first exploration of the distribution of a *Mantoniella* strain using non-metabarcoding or MAG-based means. *M. tinhauana* distribution was cosmopolitan at low level and with higher prevalence at high latitudes. Temperature and salinity were negatively correlated with *M. tinhauana* RPKM values. Nitrate concentration was the most positively correlated environmental variable, although further studies on growth rate and nutrient deficiencies would be informative. To further understand Mamiellophyceae distribution, future metagenomic studies should use expanded datasets to explore depth variations, seasonality and potentially conduct a more long-term analysis of global warming. It is important to understand the precise relationship between the environment and Mamiellophyceae dominance, as these organisms play a crucial role in ocean primary productivity. Such knowledge is essential for comprehending the dynamics of the ocean as a whole and its future implications in the context of climate change.

## Data availability statement

The datasets presented in this study can be found in online repositories. The names of the repository/repositories and accession number(s) can be found in the article/Supplementary material.

## Author contributions

ER: Writing – original draft, Writing – review & editing. YX: Writing – original draft, Writing – review & editing. CY: Writing – original draft, Writing – review & editing.
