# Supplementary material for: Genomic characterisation and ecological distribution of Mantoniella tinhauana: a novel Mamiellophycean green alga from the Western Pacific
Source: Front Microbiol. 2024 May 7;15:1358574. doi: 10.3389/fmicb.2024.1358574 (PMC11106453; doi:10.3389/fmicb.2024.1358574)
Supplement: Supplementary file 1 [file Data_Sheet_1.PDF]

Supplementary Figures

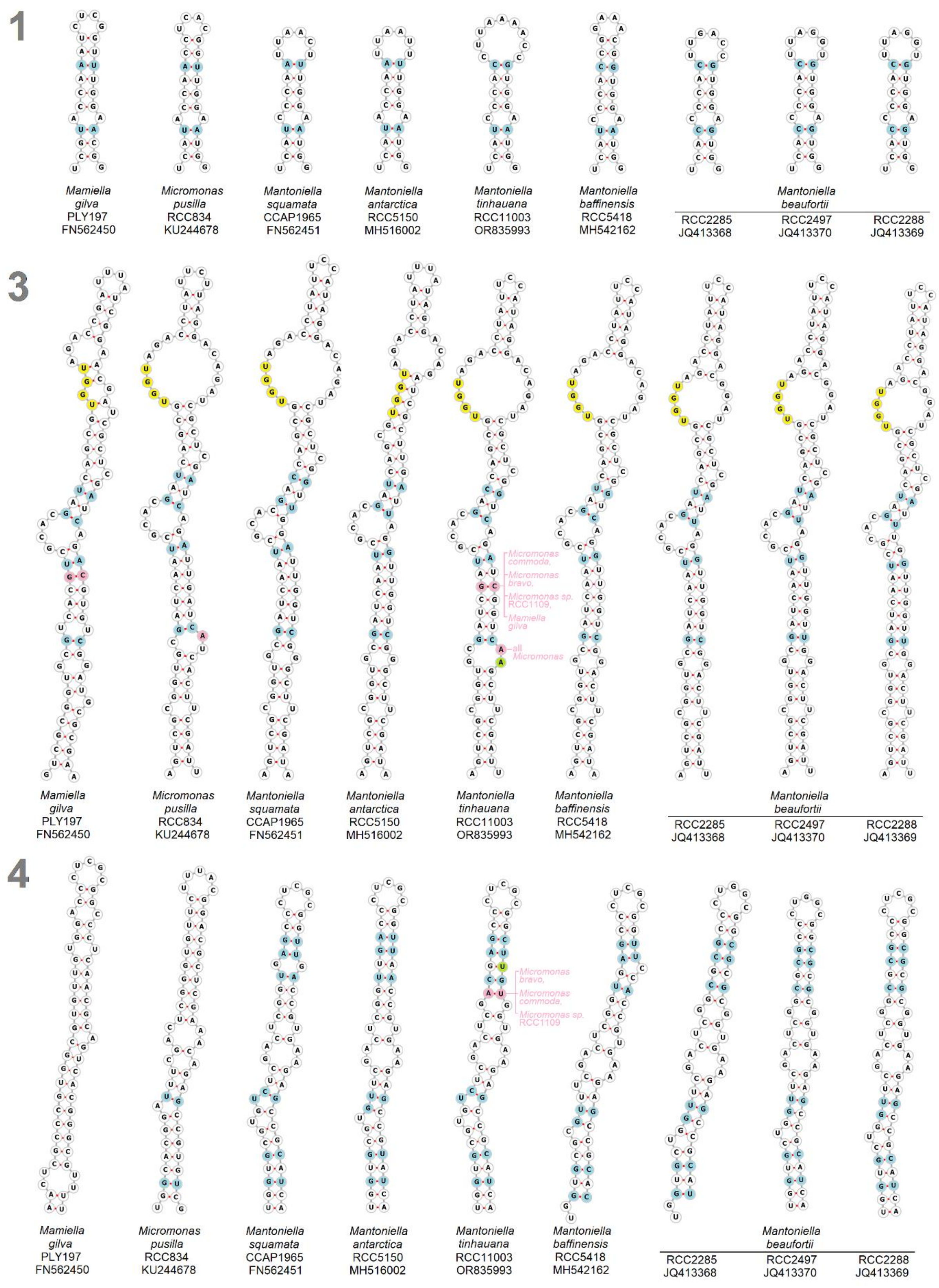

Fig. S1. ViennaRNA forna ITS2 folding structures of helices 1, 3 and 4. Base-by-base comparison of *M. tinhauana* to other *Mantoniella* and Mamiellaceae strains, displayed in ITS2-only phylogenetic order. Yellow: universal eukaryote ITS2 motifs. Blue: site of nucleotide variant present in other *Mantoniella* species with structural effect. Pink: site of nucleotide variant absent in other *Mantoniella* species, with other Mamiellophyceae species in which it is present written in pink. Green: site of nucleotide only found in the novel *M. tinhauana* strain, and no other Mamiellophyceae species.

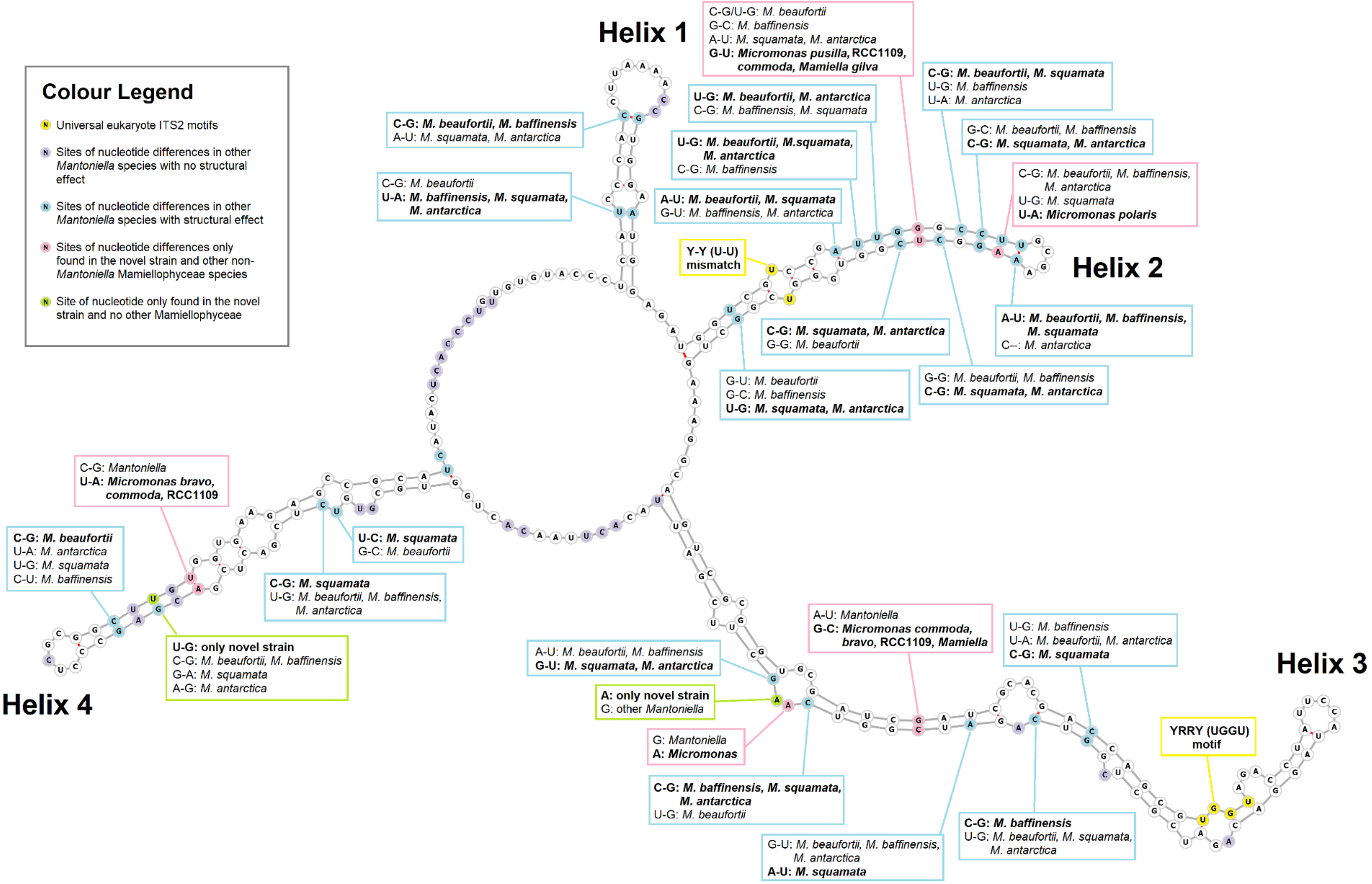

Fig. S2. Base-by-base comparison of the *Mantoniella tinhauana* ITS2 sequence and structure to other *Mantoniella* and other Mamiellophyceae species. The structure was drawn on the University of Vienna ViennaRNA web service forna and colour-edited manually based on alignment on 4SALE. The boxed labels describe what species have what sequences at each site. In each case, the pattern matching the novel *Mantoniella* sequence is in bold.
